# Structure, hydrogen bonding and thermal expansion of ammonium carbonate monohydrate

**DOI:** 10.1107/S205252061402126X

**Published:** 2014-12-01

**Authors:** A. Dominic Fortes, Ian G. Wood, Dario Alfè, Eduardo R. Hernández, Matthias J. Gutmann, Hazel A. Sparkes

**Affiliations:** a Department of Earth and Planetary Sciences, Birbeck, University of London, Malet Street, London WC1E 7HX, England; b Department of Earth Sciences, University College London, Gower Street, London WC1E 6BT, England; c Instituto de Ciencia de Materiales de Madrid, Campus de Cantoblanco, 28049 Madrid, Spain; d ISIS Facility, Rutherford Appleton Laboratory, Harwell Science and Innovation Campus, Didcot, Oxfordshire OX11 0QX, England; e School of Chemisty, University of Bristol, Bristol BS8 1TS, England

**Keywords:** ammonium carbonate, neutron diffraction, Raman spectroscopy, density functional theory

## Abstract

We have determined the crystal structure of ammonium carbonate monohydrate, (NH_4_)_2_CO_3_·H_2_O, using Laue single-crystal diffraction methods with pulsed neutron radiation. The crystal is orthorhombic, space group *Pnma* (*Z* = 4), with unit-cell dimensions *a* = 12.047 (3), *b* = 4.453 (1), *c* = 11.023 (3) Å and *V* = 591.3 (3) Å^3^ [ρ_calc_ = 1281.8 (7) kg m^−3^] at 10 K. The single-crystal data collected at 10 and 100 K are complemented by X-ray powder diffraction data measured from 245 to 273 K, Raman spectra measured from 80 to 263 K and an athermal zero-pressure calculation of the electronic structure and phonon spectrum carried out using density functional theory (DFT). We find no evidence of a phase transition between 10 and 273 K; above 273 K, however, the title compound transforms first to ammonium sesquicarbonate monohydrate and subsequently to ammonium bicarbonate. The crystallographic and spectroscopic data and the calculations reveal a quite strongly hydrogen-bonded structure (*E*_HB_ ≃ 30–40 kJ mol^−1^), on the basis of H⋯O bond lengths and the topology of the electron density at the bond critical points, in which there is no free rotation of the ammonium cation at any temperature. The barrier to free rotation of the ammonium ions is estimated from the observed librational frequency to be ∼ 36 kJ mol^−1^. The *c*-axis exhibits negative thermal expansion, but the thermal expansion behaviour of the *a* and *b* axes is ormal.

## Abstract 2

Single-crystal neutron diffraction, *ab initio* calculations and Raman spectroscopy are applied to understand the structure and hydrogen bonding of ammonium carbonate monohydrate, a hitherto poorly characterized substance, particularly in relation to other ammonium-bearing compounds.

## 1. Introduction   

**Figure 1:**
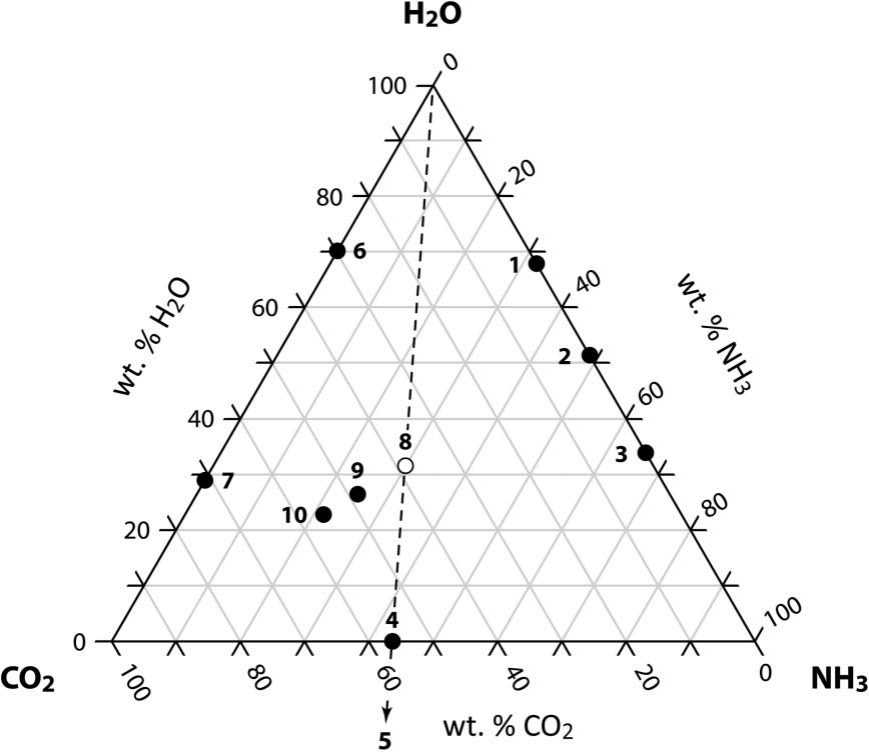
The ternary system H_2_O–CO_2_–NH_3_. With the exception of crystalline end members (*e.g.* water ice, dry ice), the compositions of compounds with known crystal structures are marked with filled circles; the only compound with an as-yet undetermined structure is the title compound, ammonium carbonate monohydrate (marked with an open circle). Numbers indicate the following phases: (1) ammonia dihydrate; (2) ammonia monohydrate; (3) ammonia hemihydrate; (4) ammonium carbamate (α and β polymorphs); (5) urea; (6) CO_2_ clathrate hydrate; (7) solid carbonic acid (α and β polymorphs); (8) ammonium carbonate monohydrate; (9) ammonium sesquicarbonate monohydrate; (10) ammonium bicarbonate.

Interactions between the most simple of molecules are of fundamental interest across diverse areas of the physical sciences, as well as underpinning a number of important industrial and biological processes; the ternary system H_2_O–CO_2_–NH_3_ is no exception. As shown in Fig. 1 ▸, many different solid phases crystallize in this system, including a number of ternary compounds, these being ammonium carbonate monohydrate [(NH_4_)_2_CO_3_·H_2_O], ammonium sesquicarbonate monohydrate [(NH_4_)_4_(H_2_(CO_3_)_3_·H_2_O] and ammonium bicarbonate [(NH_4_)_2_HCO_3_]. Since the earliest contribution to our knowledge of these materials (*e.g.* Davy, 1800 ▸) there have been numerous contradictory observations regarding both the correct composition of the solid phases (*cf.* Divers, 1870) and accurate solid–liquid phase equilibria (Terres & Weiser, 1921 ▸; Terres & Behrens, 1928 ▸; Jänecke, 1929 ▸; Guyer *et al.*, 1940 ▸; Guyer & Piechowiez, 1944 ▸, 1945 ▸; Verbrugge, 1979 ▸; Kargel, 1992 ▸), in part due to a tendency for mixtures of phases to crystallize, for equilibrium to be slow to achieve and the instability of the ‘normal’ ammonium carbonate in air. Indeed the composition of commercially available ammonium carbonate has been questioned in the scientific literature up until relatively recently (Sclar & Carrison, 1963 ▸; Kuhn *et al.*, 2007 ▸); a sample of ‘ammonium carbonate’ we purchased from Sigma-Aldrich (207861, ACS reagent, ≥ 30.0% NH_3_ basis) and stored at room temperature proved to consist principally of ammonium carbamate (NH_4_·NH_2_CO_2_). It is therefore curious that there has been so little interest in the structure and properties of these comparatively innocuous materials, considering the extensive crystallographic studies of related compounds that are either toxic (ammonium oxalate) or explosive (ammonium chlorate and ammonium nitrate). The structure of ammonium sesquicarbonate monohydrate was reported for the first time just over 10 years ago (Margraf *et al.*, 2003 ▸), whilst the last paper of any significance on the crystallography of ammonium carbonate mononhydrate was published 140 years ago (Divers, 1870); the first determination of its structure and properties form the basis of this work.

Of the compounds shown in Fig. 1 ▸, only ammonium bicarbonate is known to occur naturally on Earth, as the mineral teschemacherite. This substance is found in layers of guano in South Africa and South America (Teschemacher, 1846 ▸: Ulex, 1848 ▸: Phipson, 1863 ▸) and in geothermal waters from New Zealand (Browne, 1972 ▸). It is possible that ammonium carbonate monohydrate can also occur in the faecal deposits of marine birds in periglacial environments but has hitherto eluded discovery by virtue, amongst other things, of being unstable in air above 273 K.

Ammonium carbonate is more likely to occur as an abundant mineral outside the Earth. Carbon dioxide, ammonia and water are common in interstellar, cometary and planetary ices (Allamandola *et al.*, 1999 ▸; Mumma & Charnley, 2011 ▸; Clark *et al.*, 2013 ▸) and models have indicated that condensation of ice and ammonium carbonate should have occurred in the primitive solar nebula (Lewis & Prinn, 1980 ▸). Interaction of CO_2_ with aqueous ammonia during the accretion or differentiation of icy planetary bodies is likely to have sequestered any free ammonia in the form of solid ammonium carbonates (Kargel, 1992 ▸), and this is the leading hypothesis for the lack of any appreciable ammonia or ammonia hydrates on planetary surfaces. It is plausible, therefore, that ammonium carbonates are major ‘rock-forming’ minerals in the outer solar system. This has prompted interest in extending current Pitzer potential models to accommodate likely phase assemblages produced by freezing of aqueous ammonia- and ammonium-bearing liquids in the outer solar system (Marion *et al.*, 2012 ▸), such as may occur in subsurface seas and global oceans inside Saturn’s moons Enceladus and Titan. In the same context, ammonium carbonates may have some astrobiological relevance. Being relatively refractory (and thus stable) compared with other solar system ices, ammonium carbonates represent a potentially important reservoir of C, H, O and N that may be processed by energetic phenomena into pre-biotic molecules. For example, radiolysis of ammonium carbonate has been demonstrated to produce simple amino acids (Hartman *et al.*, 1993 ▸). Conversely, ammonium carbonate may be synthesized *in situ* on the surface of Saturn’s giant satellite Titan. It is known from laboratory analogue experiments that the organic molecules produced photochemically in Titan’s dense N_2_/CH_4_ atmosphere may be hydrolyzed in aqueous ammonia to form both urea and amino acids (Poch *et al.*, 2012 ▸). Further hydrolysis of urea would be expected to form ammonium carbonates (Clark *et al.*, 1933 ▸); on Earth, this process is mediated in soils with the aid of bacterial urease, whereafter the carbonate breaks down to ammonia and water (*e.g.* Chin & Kroontje, 1963 ▸). On Titan, meteorite impacts into the icy bedrock would provide the requisite liquid in the form of impact melt to hydrolyse any solid organics (Artemieva & Lunine, 2003 ▸, 2005 ▸) and both urea and ammonium carbonate may be substantial by-products, persisting on geological timescales at the surface temperature of 95 K.

As part of a long-term program of studying planetary ices, we are investigating the interaction of both reduced carbon and oxidized carbon with other volatiles to produce non-stoichiometric compounds (such as clathrates) and stoichiometric compounds (such as carbonates). Our aims here are:

(i) to provide data necessary to identify this material in planetary environments, whether by *in situ* X-ray diffractometry (Fortes *et al.*, 2009 ▸) or by *in situ* Raman scattering (*e.g.* Jehlicka *et al.*, 2010 ▸), and
(ii) to lay the foundations for measuring the thermoelastic properties necessary to include ammonium carbonate in structural and evolutionary models of icy planetary body interiors.

## 2. Experimental and computational methods   

### 2.1. Sample preparation, X-ray powder diffraction and indexing   

**Figure 2:**
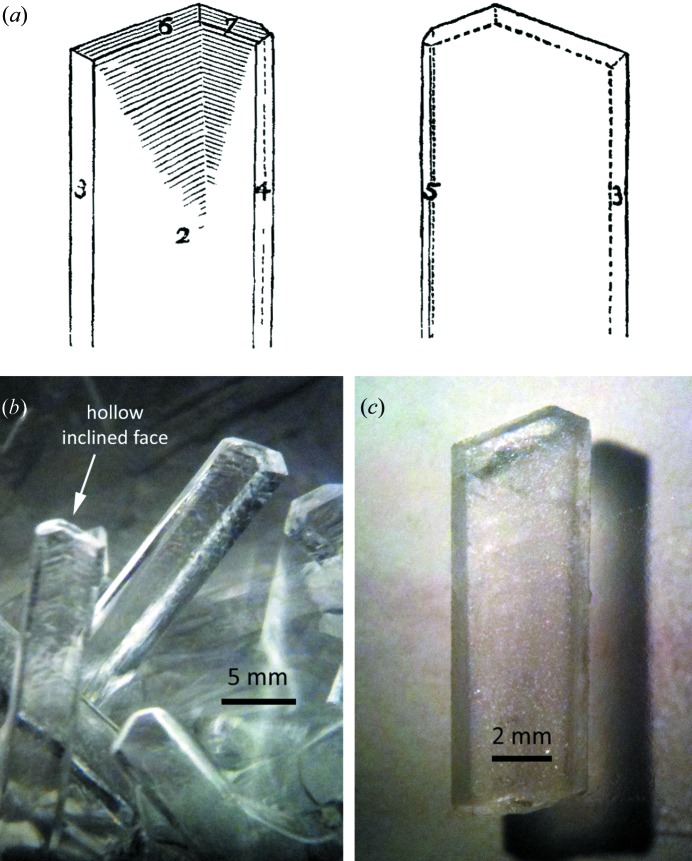
(*a*) Drawing of ammonium carbonate monohydrate crystal from Divers (1870 ▸) compared with photographs of our crystals (*b*) immersed in solution, and (*c*) in air.

Crystals were grown by exposing a beaker filled with aqueous ammonia (Sigma-Aldrich 320145 ACS reagent grade, 28–30% NH_3_) to a CO_2_-rich atmosphere inside a loosely sealed plastic bag charged with dry-ice pellets. Crystals up to several centimetres in length grew rapidly, their morphology (Fig. 2 ▸) closely resembling that described by Divers (1870 ▸). The distinct herringbone pattern and hollowed-out terminating faces remarked upon by Divers are apparent in our crystals. We established by X-ray diffraction methods that the broad face marked ‘2’ in Fig. 2 ▸(*a*) is the (0 1 0) pinacoid. Visual inspection of our microphotographs leads us to conclude that faces 3, 4 and 5 in Fig. 2 ▸(*a*) represent the orthorhombic prism form {2 1 0}, whilst faces 6 and 7 represent the {1 1 2} bipyramid. Both {1 0 0} and {1 0 2} forms occur but we have not made a detailed morphological or goniometric study. For comparison, a morphological analysis of ammonium sesquicarbonate and bicarbonate crystals is provided by Sainte-Claire Deville (1854 ▸).

Since the crystals readily lose ammonia at room temperature, all handling and characterization was carried out at low temperatures in UCL Earth Science’s Cold Room suite (air temperatures 263–253 K). The large prismatic crystals were easily extracted from the growth solvent and dried on filter paper. The initial identification of these crystals was carried out using X-ray powder diffraction methods, for which purpose the crystals were ground to a powder in a stainless-steel pestle and mortar under liquid nitrogen. The measurements were performed on our custom-made portable cold stage (Wood *et al.*, 2012 ▸); this device was pre-chilled in a chest freezer at 250 K before being loaded. The stage’s Peltier cooling device was connected to a power supply in the X-ray diffractometer enclosure within 30 s of leaving the cold room, ensuring that the specimen did not warm substantially above 253 K prior to the start of the measurement.

X-ray powder diffraction data were acquired on the cold stage at 245 K using a PANalytical X’Pert Pro diffractometer with Ge monochromated Co *K*α_1_ radiation (λ = 1.789001 Å). Comparatively weak Bragg peaks from water ice were identified, probably due to frozen mother liquor occluded within the crystals; however, the remaining peaks did not match any of the other possible known phases in the H_2_O–CO_2_–NH_3_ ternary system and no other match was found in the ICDD by the proprietary PANalytical ‘High-Score’ software. It is worth noting that the ICDD pattern described as ‘(NH_4_)_2_CO_3_·H_2_O’ with peak positions tabulated by Hanawalt *et al.* (1938 ▸; pattern no. 31), which was presumably measured at room temperature, was suggested to be a double salt of ammonium carbamate and ammonium bicarbonate by Sclar & Carrison (1963 ▸). A successful indexing of our unidentified peaks was achieved using *DICVOL*06 (Boultif & Louër, 2004 ▸), which yielded an orthorhombic unit cell of dimensions *a* = 12.129 (3), *b* = 4.480 (2), *c* = 10.998 (3) Å and *V* = 597.6 Å^3^ at 245 K. Analysis of the systematic absences in the diffraction pattern constrained the space group to one of two possibilities, *Pnma* or *Pn*2_1_ *a*.

Additional X-ray powder diffraction data were collected at 273 and 291 K by reducing the power supplied to the cold stage. One last aliquot of ammonium carbonate monohydrate crystals was crushed and allowed to sit in air at 299 K for 2 h; this material was powdered and then loaded into a standard spinner sample holder for X-ray powder diffraction analysis under air at room temperature. Powder diffraction data were analysed using the *GSAS*/*Expgui* package (Larsen & Von Dreele, 2000 ▸: Toby, 2001 ▸).

### 2.2. Neutron single-crystal diffraction and structure solution   

A single crystal of the title compound was cut into two crude cuboids in the UCL Earth Sciences cold room, one approximately 4 × 4 × 4 mm and the other roughly half the size. The crystals demonstrated an unfortunate propensity to cleave parallel to their well developed (0 1 0) faces whilst being cut. These two crystals were loaded together into a vanadium tube of 6 mm internal bore and transported to the ISIS neutron facility under liquid nitrogen. The sample canister was screwed to the end of a centre stick whilst being kept immersed in liquid nitrogen and was then transferred directly to a closed-cycle-refrigerator (equilibrated at 100 K) on the SXD beamline (Keen *et al.*, 2006 ▸). After cooling to 10 K, time-of-flight (t.o.f.) Laue data were collected in a series of five orientations, counting each for ∼ 4.5 h. The diffraction spots were indexed with the unit cell obtained at 245 K, after which the intensities were extracted using the three-dimensional profile fitting method implemented in *SXD*2001 (Gutmann, 2005 ▸).

The crystals were removed from the beamline in order to measure another specimen, and subsequently re-mounted after being stored for 2 d under L-N_2_, whereupon a second set of t.o.f. Laue data were obtained at 100 K; these were collected over five orientations, counting each for ∼ 3 h.

**Figure 3:**
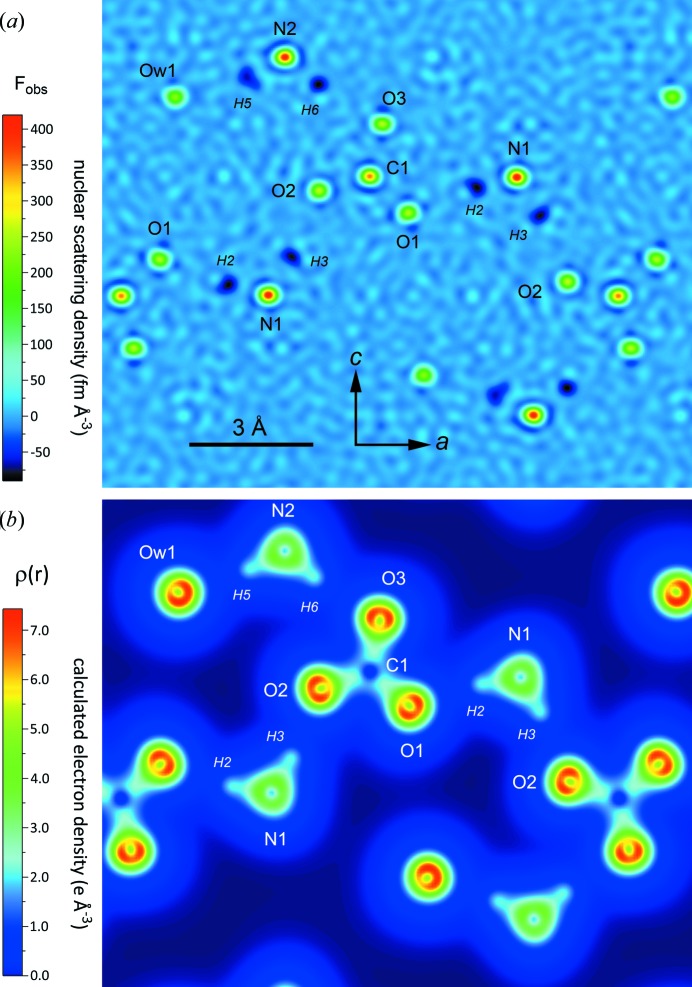
(*a*) Slice through *F*_obs_, a Fourier map computed from the observed structure factors phased on the refined atomic coordinates. The slice shows the nuclear scattering density in the *ac* plane at *b* = 0.75; positive maxima correspond to C, N and O atoms, whereas negative minima correspond to H atoms. A drawing of the structure in this plane is shown in Fig. 5 ▸. (*b*) Electron density in the same plane as Fig. 3 ▸(*a*) calculated by VASP at zero pressure and temperature. Two-dimensional visualizations created using *VESTA* (Momma & Izumi, 2011 ▸).

The 10 K data were used to solve the structure by direct methods with *SHELX*2014 (Sheldrick, 2008 ▸; Gruene *et al.*, 2014 ▸). The program *SHELXS* was used to locate positive scattering density peaks corresponding to the non-H atoms in the structure, and refinement with *SHELXL* was subsequently used to identify the residual negative peaks due to hydrogen (arising from the negative neutron scattering length of ^1^H). All atomic coordinates were refined anisotropically to yield the agreement factors listed in Table 1 ▸. The maximum and minimum peaks in the difference Fourier, Δρ = *F*_obs_ − *F*_calc_, may seem rather large in comparison to similar X-ray diffraction measurements and are better understood by reference to the ‘observed’ nuclear scattering density map, *F*_obs_, shown in Fig. 3 ▸; the difference maxima have magnitudes approximately 1% of the full range of scattering densities and are equivalent to approximately 5% of the scattering density due to a H atom. Consequently, these ‘large’ residual Fourier peaks should not be interpreted as missing atoms.

### 2.3. Raman spectroscopy   

Laser stimulated Raman spectra were measured using a portable B&WTek *i*-Raman Plus spectrometer equipped with a 532 nm laser (*P*_max_ = 37 mW at the probe tip) that records spectra over the range 168–4002 cm^−1^ with a resolution of ∼ 3 cm^−1^. Measurements were carried out on large single crystals of ammonium carbonate monohydrate in our cold room using the BC100 fibre-optic coupled Raman probe. Background noise was minimized by acquisition of multiple integrations, each of 30 to 50 s (at 50% laser power, 18 mW), the time per integration being limited by detector saturation. At 263 K spectra were integrated for a total of 600 s. Measurements made with the crystal at dry ice temperatures (195 K) or at liquid nitrogen temperatures (77 K) were integrated for 500 and 100 s, respectively.

### 2.4. Computational methods   

In order to confirm the veracity of our structure solution, and to aid in interpretation of the Raman spectrum, we carried out a first-principles calculation using density functional theory (DFT; Hohenberg & Kohn, 1964 ▸; Kohn & Sham, 1965 ▸), as implemented in the Vienna *Ab initio* Simulation Package, VASP (Kresse & Furthmüller, 1996 ▸). The plane-wave expansion was treated using the projected augmented-wave method, PAW (Blöchl, 1994 ▸); with the PAW potentials generated by Kresse & Joubert (1999 ▸) and distributed with VASP. The exchange-correlation was accommodated using the PBE generalized gradient corrected functional (Perdew *et al.*, 1996 ▸, 1997 ▸). This form of the generalized gradient approximation (GGA) has been demonstrated to yield results of comparable accuracy to higher-level quantum chemical methods, such as MP2 and coupled-cluster methods, in hydrogen-bonded systems (*e.g.* Ireta *et al.*, 2004 ▸), despite not correctly representing dispersion forces.

Convergence tests were carried out to optimize the **k**-point sampling of the Brillouin zone within the Monkhorst–Pack scheme (Monkhorst & Pack, 1976 ▸) and the kinetic energy cut-off of the plane-wave basis set. It was found that a 2 × 5 × 2 **k**-point grid combined with a kinetic energy cut-off of 944 eV yielded a total-energy convergence better than 10^−3^ eV per atom and pressure converged better than 0.2 GPa. A structural relaxation under zero-pressure athermal conditions was carried out, starting from the experimental crystal structure obtained at 10 K, in which the ions were allowed to move according to the calculated Hellman–Feynman forces and the unit-cell shape was allowed to vary. The relaxation was stopped when the forces on each atoms were less than 5 × 10^−4^ eV Å^−1^ and each component of the stress tensor was smaller than 0.05 GPa. The phonon spectrum was then computed using the small displacement method as implemented in the PHON code (Alfè, 2009 ▸). The construction of the full force-constant matrix requires knowledge of the force-field induced by displacing each atoms in the primitive cell in the three Cartesian directions. Since there are 68 atoms in the primitive cell, the total number of required displacements for this system would then be 408 (allowing for both positive and negative displacements), although this can be reduced to 84 by exploiting the symmetry elements present in the crystal. We used displacements of 0.01 Å, which are sufficiently small to obtain phonon frequencies that are converged to better than 0.1%. Since, in this instance, we are interested only in the normal modes at the Brillouin zone (BZ) centre, all of the required information could be obtained by computing the force matrix for the primitive unit cell. However, in order to check the mechanical stability of the compound we also computed the force constant matrix using a 2 × 5 × 2 supercell, which is large enough to provide fully converged phonon frequencies in the whole BZ, and we found no imaginary phonon branches.

The resulting modes at the BZ centre were classified according to the irreducible species of point group *D*_2*h*_; this was done using standard group theory techniques with the help of the program *SAM* (Kroumova *et al.*, 2003 ▸), available at the Bilbao crystallographic server (Aroyo *et al.*, 2006 ▸).

## 3. Results and discussion   

### 3.1. Description of the structure and bonding   

**Figure 4:**
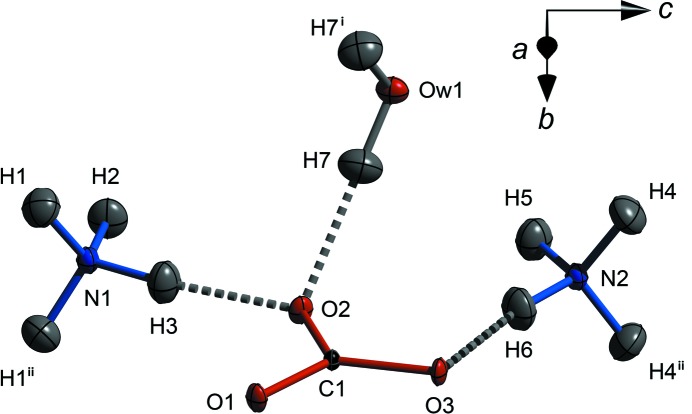
The asymmetric unit of ammonium carbonate monohydrate with atomic displacement ellipsoids determined at 100 K drawn at the 50% probability level. Dashed rods correspond to hydrogen bonds. Superscripts denote symmetry operations (i) 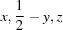; (ii) 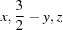. Three-dimensional structure visualizations created using *DIAMOND* (Putz & Brandenburg, 2006 ▸).

Fig. 4 ▸ depicts the asymmetric unit with atoms labelled according to the scheme employed in all subsequent figures and tables. In addition to the tabulated data presented here, we have deposited supplementary CIFs containing all structure factors (*hkl*), refinement output (*SHELX* RES) and interatomic distances and angles.^1^ The results of the athermal zero-pressure DFT structural relaxation are in close agreement with the observed structure (see CIF and Table S1 in the supporting information).

The CO_3_^2−^ anions have trigonal planar symmetry (point group *D*_3*h*_) within experimental error and lie in planes perpendicular to the *b*-axis at *y* = 0.25 and 0.75. Each of the carbonate O atoms accepts three hydrogen bonds, one each in the plane of the anion and two out-of-plane. The in-plane hydrogen bonds are donated exclusively by neighbouring ammonium cations, two from the N(1)H_4_^+^ unit, which form chains of NH_4_^+^—CO_3_^2−^—NH_4_^+^ extending along the *a*-axis (Figs. 3 ▸ and 5 ▸), and one from the N(2)H_4_^+^ unit. For O1 and O3, the out-of-plane hydrogen bonds are donated by NH_4_^+^ tetrahedra in adjacent planes, whereas O2 accepts hydrogen bonds from the water molecule in adjacent planes. The in-plane hydrogen bonds from N(1)H_4_^+^ cations that form the *a*-axis chains are significantly shorter (mean = 1.748 Å) than the other in- and out-of-plane hydrogen bonds (mean = 1.809 Å). As shown in Fig. 6 ▸, chains in adjacent sheets are related to one another by the 2_1_ symmetry operation along *b*, the result being a fully three-dimensional hydrogen-bonded framework, albeit with a strongly layered character. Note that the crystals (Fig. 2 ▸) are elongated in the direction of these strongly hydrogen-bonded chains and the layering is parallel to the broad (0 1 0) faces.

The only substantial difference between the experimental and computational structures is in the length and linearity of the hydrogen bonds donated by and accepted by the water molecule. In the DFT relaxation, the hydrogen bond accepted from N(2)H_4_^+^ is approximately 4.5% shorter and ∼ 5° straighter than is observed in the experimental data. The hydrogen bond donated from H_2_O to the O2 carbonate oxygen is ∼ 1.8% shorter than we see experimentally; this bond is already almost perfectly linear in both the computational and experimental data. Note that the other calculated hydrogen-bond lengths are just a few tenths of a percent different from the single-crystal structure refinements, differences that have little or no statistical significance. See Tables 2–4 ▸ ▸ ▸.

The carbonate ions are entirely unremarkable, having nearly identical C—O bond lengths to those found by single-crystal methods in a large number of other inorganic carbonate minerals (Zemann, 1981 ▸; Hesse *et al.*, 1983 ▸; Chevrier *et al.*, 1992 ▸; Maslen *et al.*, 1995 ▸; Giester *et al.*, 2000 ▸) and in ammonium sesquicarbonate monohydrate (Margraf *et al.*, 2003 ▸).

By contrast, there are fewer examples of accurate N—H bond lengths for ammonium ions in the literature obtained by single-crystal neutron diffraction methods. Moreover, these ions appear to be more susceptible than the carbonate ion to variations in bond length depending on the coordination environment (Brown, 1995 ▸; Demaison *et al.*, 2000 ▸). Table 5 ▸ lists a number of experimental and computational values for the N—H (or N—D) bond length of the ammonium ion in the gas phase, in clusters and in crystals (for which we report only single-crystal neutron diffraction data).

The equilibrium N—H bond length for the gas phase NH_4_^+^ ion has been determined spectroscopically to be 1.029 Å (Crofton & Oka, 1987 ▸), and many of the crystallographic values fall around this value; indeed the mean of all values listed in Table 5 ▸ is 1.026 Å. One would expect the N—H contact to increase in length on formation of hydrogen bonds, and there is some computational support for this; Jiang *et al.* (1999 ▸) reported a small increase in *r*(N—H) on formation of hydrogen-bonded clusters with water, and in our own earlier work (Fortes *et al.*, 2001 ▸) we found that *r*(N—H) increased from 1.055 Å in the free ion to 1.063 Å in the hydrogen-bonded NH_4_OH crystal. Nonetheless, we still see long N—H bonds (> 1.05 Å) even in weakly hydrogen-bonded crystals, such as ammonium perchlorate (Choi *et al.*, 1974 ▸), where the ammonium ions have a very low energy barrier to free rotation, ∼ 2 kJ mol^−1^ (Johnson, 1988 ▸; Trefler & Wilkinson, 1969 ▸; Westrum & Justice, 1969 ▸).

A more accurate picture of the covalent N—H bond in the ammonium ion may be obtained instead by analysis of the electron density generated by DFT calculations. Fig. 3 ▸(*b*) depicts a slice through the crystal in the plane of the N1—H2, N1—H3, N2—H5 and N2—H6 bonds. The properties of these bonds are described by the topology of the electron density according to Bader’s quantum theory of atoms in molecules, QTAIM (Bader, 1990 ▸). Of interest to us are the saddle points where the gradient in the electron density, ∇ρ(*r*), vanishes; these are known as bond critical points (BCPs). Important metrics of the bond strength and character are the electron density at the BCP, ρ(*r*_BCP_), and the Laplacian of the electron density at the BCP, ∇^2^ρ(*r*_BCP_), which itself represents the 3 × 3 Hessian matrix of second partial derivatives of the electron density with respect to the coordinates. The eigenvalues of this matrix, λ_1_, λ_2_ and λ_3_ (which sum to ∇^2^ρ) are the principal axes of ‘curvature’ of the electron density perpendicular to the bond (λ_1_, λ_2_) and along the bond (λ_3_). At the bond critical points these eigenvalues have different signs, which may lead (particularly for weak bonds, as we will see later) to Laplacians with small values and comparatively large uncertainties. Indeed it has been shown by Espinosa *et al.* (1999 ▸) that the curvature along the bond, λ_3_, provides the clearest indicator of bond strength. Nonetheless, the Laplacian is still a widely reported quantity; a negative Laplacian at the bond critical point generally corresponds to a concentration of electron density, which is characteristic of a covalent bond, whereas ionic bonds and hydrogen bonds have a positive Laplacian, indicative of a depletion in electron density.

We have used the program *AIM-UC* (Vega & Almeida, 2014 ▸) to compute the properties of the electron density at the N—H bond critical points in ammonium carbonate monohydrate (Table 6 ▸). For comparison we have reproduced some experimental and computational electron density metrics from other ammonium compounds, revealing some interesting differences. Note that the density at the BCP is broadly similar for all compounds, but the Laplacians range from approximately −10 e Å^−5^ (NH_4_F) to −50 e Å^−5^ (this work). Between the four literature examples (all with N—H bond lengths of ∼ 1.030 Å), much of the difference lies in the value of λ_1_ and λ_2_; however, for ammonium carbonate monohydrate the greatest difference is in λ_3_, which has a value of ∼ 10 e Å^−5^, relative to 20–30 e Å^−5^ in the other materials. It is intriguing that such significant differences in charge distribution should exist within otherwise similar ionic entities.

Of the two symmetry-inequivalent NH_4_^+^ cations only the N2 unit bonds with the water molecule, although this appears to have little effect on either the N—H or the H⋯O bond between the ammonium ion and the water molecule compared with any other interatomic contact. The water molecules themselves are, however, somewhat unusual in being trigonally coordinated rather than tetrahedrally coordinated as one might expect, accepting just a single hydrogen bond from the H5 atom. However, this is not unprecedented; a similar arrangement occurs in a number of inorganic hydrates, such as ammonia dihydrate (Loveday & Nelmes, 2000 ▸; Fortes *et al.*, 2003 ▸).

Following the example outlined above for the ammonium ion, we can also use the QTAIM methodology to characterize the hydrogen-bond network that holds together each of the material’s molecular and ionic building blocks. Based solely on the H⋯O distances and N—H⋯O angles, it is clear that ammonium carbonate monohydrate is relatively unusual amongst ammonium compounds in having quite strong hydrogen bonds donated by the ammonium ion. The effect of this on the vibrational frequencies is also quite clear as described in the following section. As before, we used *AIM-UC* to obtain the coordinates and topological properties of the bond critical points relating to the hydrogen bonds and these are listed in Table 7 ▸. There are well known correlations between interatomic distances and the electron density metrics (particularly λ_3_), and our results are in good agreement with previous experimental and computational values (Espinosa *et al.*, 1999 ▸; Tang *et al.*, 2006 ▸).

The dissociation energy of the hydrogen bond may be estimated accurately from vibrational frequencies and with varying degrees of accuracy from the electron density (*cf.* Vener *et al.*, 2012 ▸). The total energy density is the sum of the local kinetic and potential electronic energies, *G*(*r*) and *V*(*r*), respectively, at the BCP (Bader & Beddall, 1972 ▸)
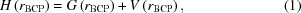
where the potential energy is related to the Laplacian of the electron density *via* the local form of the virial theorem (Bader, 1990 ▸)
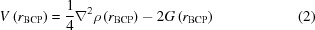
and the kinetic energy is obtained by partitioning of the electron density (*e.g.* Abramov, 1997 ▸)
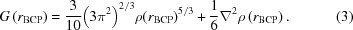
Espinosa *et al.* (1998 ▸) proposed that the hydrogen-bond energy, *E*_HB_, could be obtained simply from the potential energy density
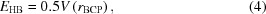
and this expression continues to be used widely, whereas Mata *et al.* (2011 ▸) subsequently suggested that a more accurate value could be found from the kinetic energy density
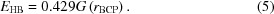
A subsequent analysis by Vener *et al.* (2012 ▸) found that equation (4) systematically overestimates *E*_HB_ compared with the spectroscopically determined hydrogen-bond energies. However, the value given by equation (5) appears to yield reasonably accurate values of *E*_HB_. In Table 8 ▸ we detail *E*_HB_ as calculated using equations (4) and (5). Furthermore, we give a ‘corrected’ value of *E*_HB_ based on equation (4) and the tabulated results in Vener *et al.* (2012 ▸) such that *E*_HB_(corrected) = 0.465*E*_HB_ + 16.58. The mean values of *E*_HB_ in the right-hand column thus represent our most accurate determination of the hydrogen-bond dissociation energy in this compound.

The arrangement of the ions in ammonium carbonate monohydrate differs in a number of important ways from related structures. As noted above, the structure consists of chains of alternating hydrogen-bonded cations and anions; *i.e.* the ions are co-planar in the plane of the carbonate ion and it is this co-planarity that is unusual. In the structures of ammonium sesquicarbonate monohydrate (Margraf *et al.*, 2003 ▸) and ammonium bicarbonate (Pertlik, 1981 ▸; Zhang, 1984 ▸) the anions are also arranged into chains (Fig. 7 ▸), but these are hydrogen bonded by water molecules and/or the O—H moiety of the bicarbonate ion. Unlike ammonium carbonate monohydrate, the cations in these two structures are situated *between* the planes of carbonate anions.

There are comparatively few other structures with which to form a direct comparison of the title compound. Of the alkali metals for which the ammonium ion most commonly substitutes, Li, Rb and Cs form poorly soluble carbonates and no hydrates are known (Dinnebier *et al.*, 2005 ▸); potassium is known only to form a sesquihydrate, K_2_CO_3_·3/2H_2_O (Skakle *et al.*, 2001 ▸). Conversely, Na_2_CO_3_ is highly soluble in water and can crystallize as a decahydrate (the mineral natron), a heptahydrate and a monohydrate (Wu & Brown, 1975 ▸).

Due to the much smaller ionic radius of Na^+^ relative to NH_4_^+^ the structure of sodium carbonate monohydrate differs in some important respects from that of ammonium carbonate monohydrate. In Na_2_CO_3_·H_2_O (space group *Pca*2_1_) there are perfectly trigonal CO_3_ anions lying in planes perpendicular to the crystal’s *a*-axis at *x* = 0.25 and 0.75. Where this structure differs from the ammonium analogue is that carbonate anions in adjacent sheets lie directly above one another (rather than being offset by half a unit cell) to form closely packed ‘columns’ of CO_3_ along the *a*-axis. The structure also differs in having both the cations and the water molecule lying in discrete sheets between the CO_3_ planes, much like ammonium sesquicarbonate and bicarbonate. The higher-density packing results in the helical structure shown in Fig. 8 ▸, linked together by water molecules in distorted tetrahedral coordination; by comparison, the ammonium carbonate crystal has simple zigzag chains linked by trigonally coordinated water molecules donating almost perfectly linear hydrogen bonds.

Amongst other possible analogues, ammonium nitrate and ammonium chlorate have no known hydrates: ammonium sulfite occurs as a monohydrate but this structure is characterized by non-planar (and non-co-planar) SO_3_ anions (Battelle & Trueblood, 1965 ▸) and so there is little to be gained by discussing this further. Only two other compounds are of any possible interest, these being ammonium carbonate peroxide (Medvedev *et al.*, 2012 ▸) and ammonium oxalate monohydrate (Robertson, 1965 ▸; Taylor & Sabine, 1972 ▸). In both of these compounds the (roughly) planar anions are linked in-plane by water or hydrogen peroxide rather than the ammonium cations. In summary, there are no obvious structural analogues amongst any plausible related compounds and the occurrence of co-planar anions and cations in these types of structures seems to be uncommon.

### 3.2. Vibrational spectra   

Ammonium carbonate monohydrate crystallizes in the centrosymmetric space group *Pnma* having a primitive cell with *D*_2*h*_ point-group symmetry and four formula units per unit cell; all ions and molecules are located on sites of *C_S_* [σ(*xz*)] symmetry. Based on a consideration of the normal vibrational modes of the free ammonium and carbonate ions and the neutral water molecule, we have carried out a factor group analysis by the correlation method to determine the symmetry species of all Raman-active modes. Allowing for the modes corresponding to translation of the entire crystal (2*A*′ + *A*″), we find that there are 102 normal modes summarized as Γ_opt_(Raman) = 31*A_g_* + 20*B*_1*g*_ + 31*B*_2*g*_ + 20*B*_3*g*_. The DFT-calculated frequencies of these 102 normal modes are shown in relation to the observed Raman spectrum in Fig. 9 ▸ ▸.

The Raman spectrum is dominated by strong symmetric stretching modes from the carbonate ion (split into a strong *A_g_* symmetry peak at 1074 cm^−1^ and a weaker *B*_2*g*_ symmetry peak at 1056 cm^−1^), from the ammonium ions (2889 cm^−1^) and from the water molecule (3297 cm^−1^). These are in good agreement with the calculated frequencies, 1044.5, 1008.3, 2920 (average) and 3343 (average) cm^−1^, respectively.

Moderately strong Raman peaks occur in the low-frequency range, between 218 and 280 cm^−1^, which are due to translational motions of the ammonium ions, and in the high-frequency range (2600–3000 cm^−1^) due to the asymmetric N—H stretch of NH_4_^+^. The latter are divided into motions in the crystal’s mirror plane (on the low-frequency side of ν_1_ NH_4_^+^) and motions out of the mirror plane (on the high-frequency side of ν_1_ NH_4_^+^). Amongst these modes, peaks due to the N(1)H_4_^+^ tetrahedron occur at lower frequency than those due to N(2)H_4_^+^, which is likely to reflect the influence of the shorter (*i.e.* stronger) hydrogen bonds donated by N(1)H_4_^+^ (see, for example, Tables 4 ▸ and 8 ▸). Fig. 10 ▸ illustrates a deconvolution of the high-frequency portion of the spectrum into separate Lorentzian contributions.

In the range 1300–1850 cm^−1^ (Fig. 11 ▸) we observe the asymmetric stretch of the carbonate ion, split into *A_g_* and *B*_2*g*_ peaks at 1385 and 1424 cm^−1^, the asymmetric deformation of the NH_4_^+^ ion between 1475 and 1555 cm^−1^, and the symmetric deformation of the NH_4_^+^ ions (scissor and twist modes) from around 1696 to 1769 cm^−1^. The symmetric bending mode of the water molecule is sometimes observed as a very weak feature near 1650 cm^−1^ [ν_2_(*A_g_*) at 1637.7 cm^−1^ and ν_2_(*B*_2*g*_) at 1636.8 cm^−1^ according to our DFT calculations], but this seems to be absent in our measured spectra. It is conceivable that the peak at 1696.1 cm^−1^ is due to this vibrational mode, but the arguments concerning combination bands made below suggest that this is not the case.

Between 300 and 1000 cm^−1^ there are some weak features, the first (from ∼ 507 to 608 cm^−1^) being due to librational motion of the NH_4_^+^ ions. A doublet at 687.6 and 709.2 cm^−1^ is attributable to asymmetric bending of the carbonate ion, whilst the peak at 745 cm^−1^ is probably due to libration of the water molecules.

The frequencies of normal modes attributed to the carbonate ion are in excellent agreement with literature data for numerous other carbonate compounds (*e.g.* Buzgar & Apopei, 2009 ▸). However, the positions of the NH_4_^+^ stretching modes are substantially red-shifted, and those of the bending modes substantially blue-shifted, from the observed vibrational frequencies of the free ion and of ammonium in weakly hydrogen-bonded solids (see Brown, 1995 ▸, and references therein). The pattern of shifts is further evidence of the relatively strong hydrogen bonding of the ammonium ion. Indeed the observed vibrational frequencies are similar to those observed in one of most strongly hydrogen-bonded ammonium salts, NH_4_F (Plumb & Hornig, 1955 ▸).

Further observational support for strong hydrogen bonding of the ammonium ion (in addition to the electron-density analysis in the previous section) is the occurrence of bands around 2000 and 2200 cm^−1^ due to the combination of NH_4_^+^ bending modes (ν_2_ and ν_4_) with the librational modes (ν_6_). Their presence is strongly supportive of rotational hindrance caused by hydrogen bonding. Since the strongest NH_4_^+^ librational peak is at 507 cm^−1^, we have simply red-shifted the combination bands by a uniform 500 cm^−1^ in Fig. 12 ▸ in order to compare their structure with the ν_2_ and ν_4_ regions. Note the occurrence of the 1696 cm^−1^ peak in the combination bands, suggesting that it is due to ammonium rather than water. Finally, the frequency of the ammonium librational mode is much higher than in many other ammonium salts; using the empirical relationship of Sato (1965 ▸) we use the observed librational frequency to derive a barrier to free rotation of 36 kJ mol^−1^. Not only is this large (*cf.* Johnson, 1988 ▸), being comparable to NH_4_F (44 kJ mol^−1^), but it is strikingly similar to the mean hydrogen-bond energy reported at the bottom of Table 8 ▸.

Inelastic neutron spectra of ‘ammonium carbonate’ were measured at 293 K by Myers *et al.* (1967 ▸); they determined the frequencies of the translational and torsional modes as, respectively, 205 ± 7 and 445 ± 10 cm^−1^. Whilst it is unlikely that the material was ammonium carbonate monohydrate in the form studied by us, their work does note that the frequency ratio of the two modes for a number of ammonium salts lies between 1.99 and 2.38, which is in good agreement with our observations.

### 3.3. Thermal expansion and decomposition   

Since we have collected crystallographic data at several temperatures (albeit on different instruments using different methods) we are in a position to make some initial remarks concerning the magnitude and anisotropy of the thermal expansion and the behaviour of the material on warming above 273 K. A more detailed experimental study of these phenomena is planned. Table 10 ▸ gives the unit-cell parameters at 10 and 100 K determined from the single-crystal neutron experiment, and at 245 K as determined by powder X-ray diffraction.

**Figure 5:**
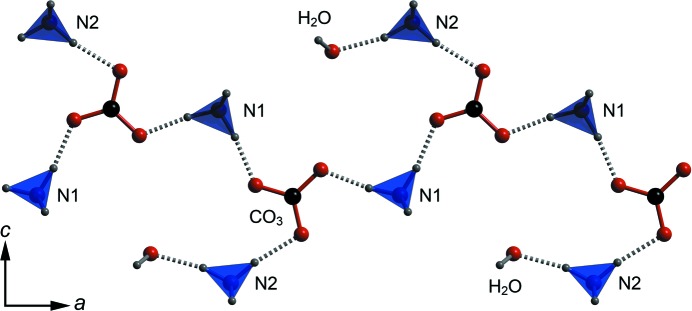
The chain motif of alternating hydrogen-bonded carbonate and N1 ammonium ions parallel to the *a*-axis. Note that the N2 ammonium cations ‘decorate’ this chain, donating the sole hydrogen bond to the water molecule. This viewing direction corresponds to that shown in Figs. 3 ▸(*a*) and (*b*). As in Fig. 4 ▸, dashed rods depict hydrogen bonds.

**Figure 6:**
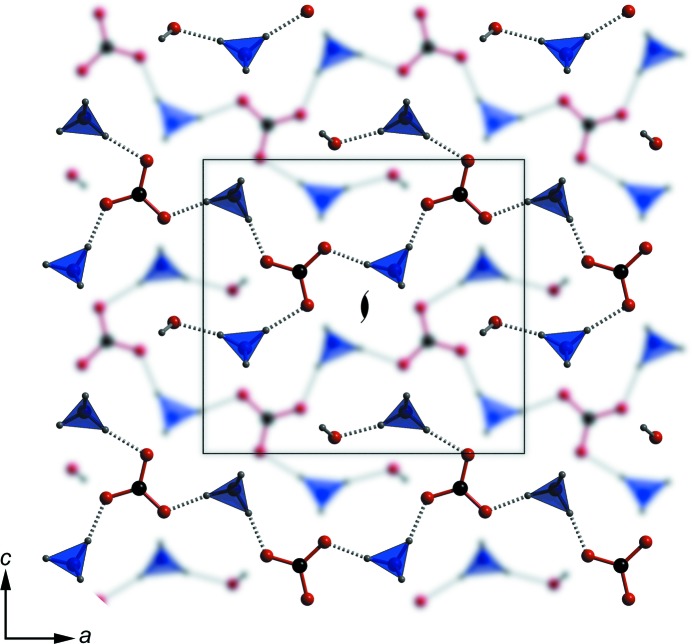
The complete structure of ammonium carbonate monohydrate is built from the chains shown in Fig. 5 ▸ arranged into sheets at *y* = 0.25 (blurred) and *y* = 0.75 (sharp). Hydrogen bonds (not shown) extend between these sheets to form a three-dimensional framework. The unit cell in the *ac* plane is marked by the superimposed box with the 2_1_ symmetry axis indicated by a symbol.

**Figure 7:**
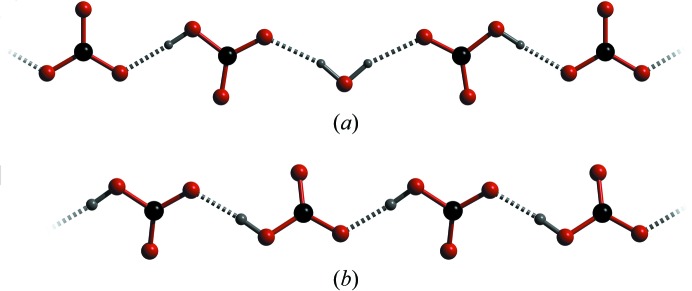
Comparison of the chain motifs occurring in (*a*) ammonium sesquicarbonate monohydrate and (*b*) ammonium bicarbonate.

**Figure 8:**
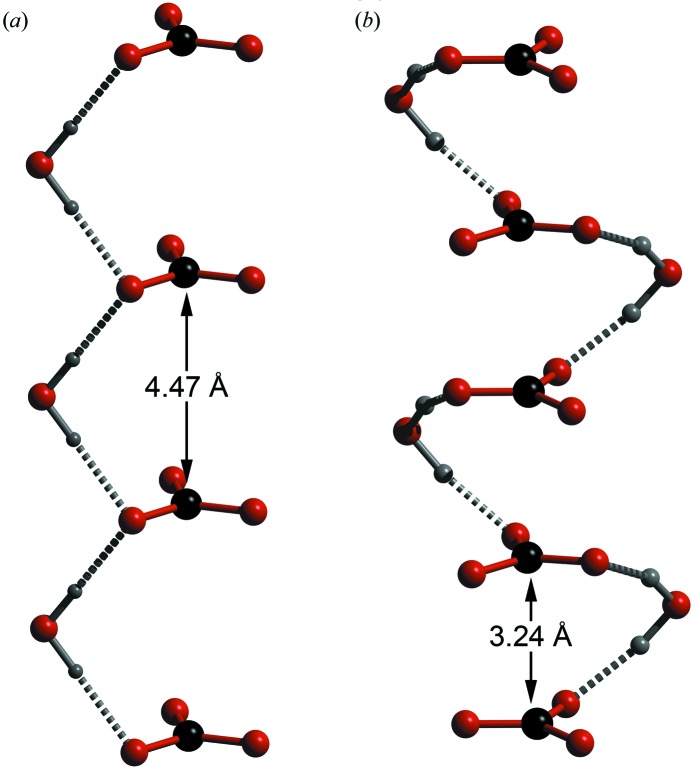
Comparison of the water–carbonate structures formed in (*a*) ammonium carbonate monohydrate and (*b*) sodium carbonate monohydrate.

**Figure 9:**
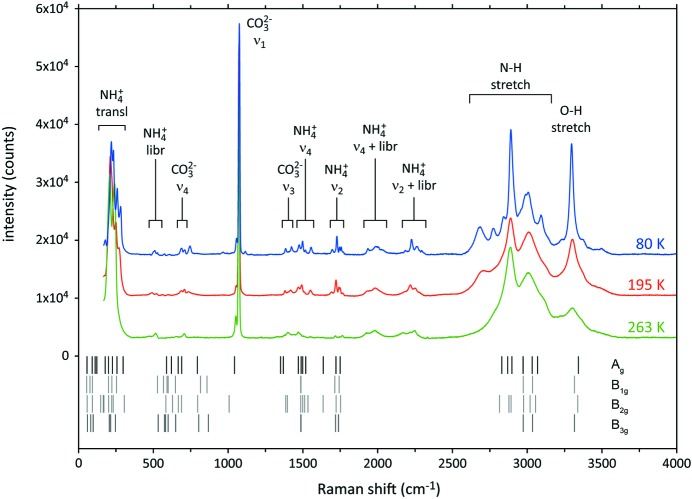
Raman spectra of ammonium carbonate monohydrate collected at 263 K (bottom), 195 K (middle) and 80 K (top). DFT-calculated Raman-active zone-centre phonon frequencies of various symmetries (*A_g_*, *B*_1*g*_, *B*_2*g*_ and *B*_3*g*_) are shown by vertical tick marks beneath the spectra. Broad identifications of the normal modes and combinations responsible for the observed bands are labelled. Additional details are given in Table 9 ▸ and Figs. 10–12 ▸ ▸ ▸.

**Figure 10:**
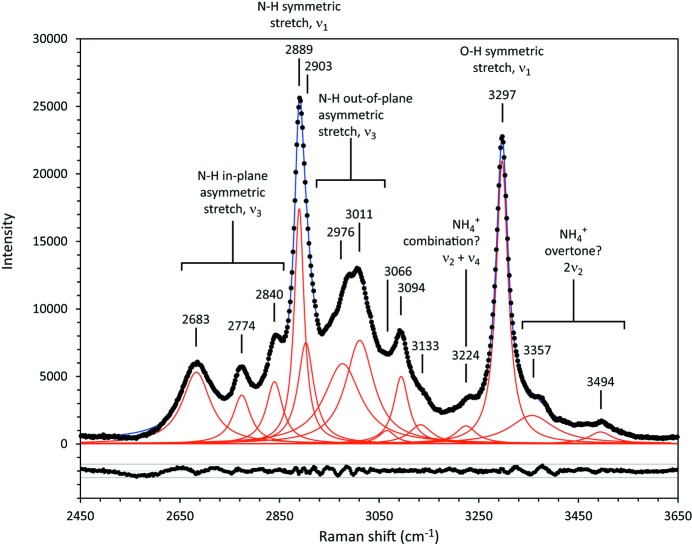
Deconvolution of the high-frequency bands and interpretation in terms of the normal modes, overtones and combinations responsible. Individual pure Lorentzian contributions are drawn in red whilst the sum is drawn in blue. Band centres are obtained by least-squares fitting. The scatterplots underneath the spectrum reports the residuals between the best fit and the data.

**Figure 11:**
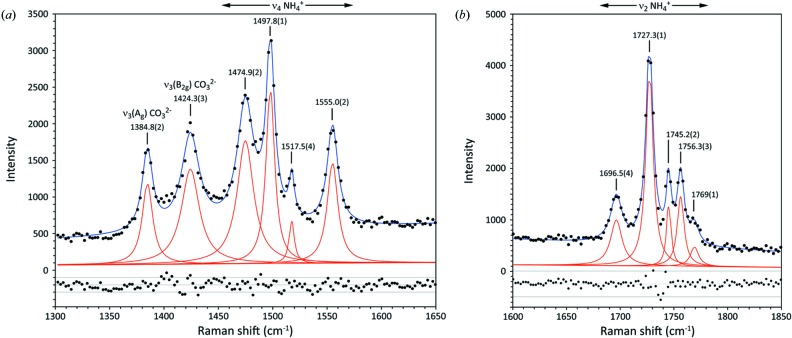
Deconvolution of the mid-frequency bands and interpretation in terms of the normal modes responsible. Individual pure Lorentzian contributions are drawn in red whilst the sum is drawn in blue. Band centres and 1σ uncertainties are obtained by least-squares fitting. The scatterplots underneath the spectra report the residuals between the best fit and the data.

**Figure 12:**
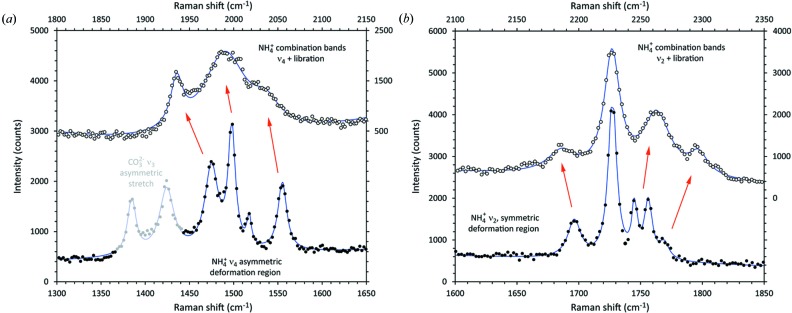
Comparison between the ammonium symmetric and asymmetric bending regions of the spectrum (*cf.* Fig. 11 ▸) and their associated bands due to combination with the librational mode. The upper sets of data have been red-shifted equally by 500 cm^−1^. The carbonate stretching modes in (*a*) are coloured grey for clarity.

**Figure 13:**
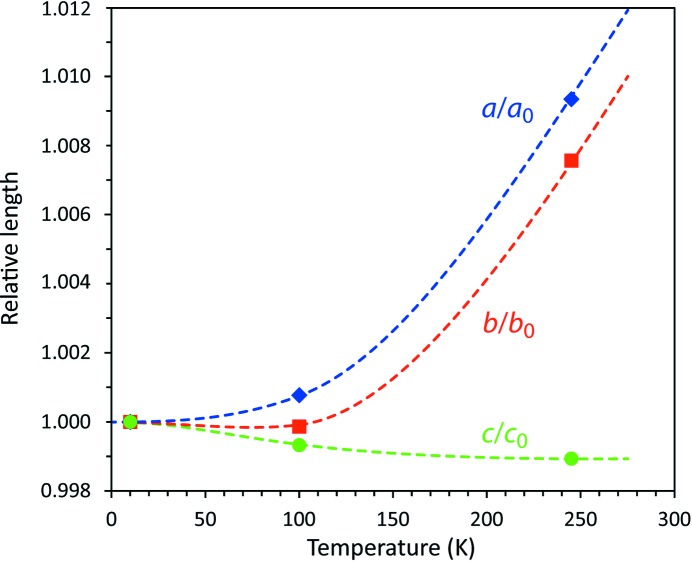
Relative thermal expansion of the three crystallographic axes in ammonium carbonate monohydrate. Symbols report data measured by neutron or X-ray diffraction and the dashed lines show qualitative interpolations (guides to the eye).

These data are illustrated in Fig. 13 ▸, which includes a qualitative representation of the anisotropic thermal expansion in the form of dashed lines, effectively no more than a visual guide. Both the *a*-axis and the *b*-axis of the crystal expand normally on warming (although the *b*-axis may exhibit a small amount of negative expansion below 100 K), whereas the *c*-axis contracts. Estimates of the thermal expansivities along the three orthogonal directions at 245 K are α*_a_* = α*_b_* ≃ 80 × 10^−6^ K^−1^ and α*_c_* ≃ −1 × 10^−6^ K^−1^, resulting in a volume thermal expansion of approximately 160 × 10^−6^ K^−1^. In other words, upon warming the crystal experiences the greatest expansion parallel to the chains drawn in Fig. 5 ▸ whilst undergoing a small degree of contraction perpendicular to those chains. The volume coefficient of thermal expansion is similar to that of water ice Ih at the same temperature (Röttger *et al.*, 1994 ▸).

X-ray powder diffraction data reveal that ammonium carbonate monohydrate begins to transform to ammonium sesquicarbonate monohydrate at around 273 K; this transformation is complete in under an hour at 291 K. After being left in air at 299 K for 2 h, we found that crushed single crystals of ammonium carbonate monohydrate had transformed completely to ammonium bicarbonate.

## 4. Summary   

We have determined the crystal structure of ammonium carbonate monohydrate, a substance that we find to be stable in air only at temperatures below 273 K, explaining why this otherwise ubiquitous laboratory reagent has been consistently misidentified over the decades. Unlike the related sesquicarbonate and bicarbonate of ammonia, the ‘normal’ carbonate consists of co-planar chains of strongly hydrogen-bonded NH_4_^+^ and CO_3_^2−^. This moderately strong hydrogen bonding results in a barrier to free rotation of the ammonium ion second only to ammonium fluoride. The structural architecture is manifested in highly anisotropic linear thermal expansion coefficients, including a negative expansivity along the *c*-axis of the crystal. Undoubtedly the material will exhibit a large elastic anisotropy, like the similarly layered ammonium oxalate monohydrate (Küppers, 1972 ▸) and we predict that the *a*-axis will prove to be the most compressible direction in the crystal whilst the *c*-axis will be the least compressible.

## Supplementary Material

Crystal structure: contains datablock(s) I, II. DOI: 10.1107/S205252061402126X/eb5035sup1.cif

Structure factors: contains datablock(s) I. DOI: 10.1107/S205252061402126X/eb5035Isup2.hkl

Structure factors: contains datablock(s) II. DOI: 10.1107/S205252061402126X/eb5035IIsup3.hkl

Supporting information file. DOI: 10.1107/S205252061402126X/eb5035Isup4.cml

Supporting information file. DOI: 10.1107/S205252061402126X/eb5035IIsup5.cml

Extra table. DOI: 10.1107/S205252061402126X/eb5035sup6.pdf

CCDC references: 1025925, 1025926

## Figures and Tables

**Table 1:** Experimental details

	10K	100K
Crystal data		
Chemical formula	CO_3_H_2_O2H_4_N	CO_3_H_2_O2H_4_N
*M*_r_	114.11	114.11
Crystal system, space group	Orthorhombic, *P* *n* *m* *a*	Orthorhombic, *P* *n* *m* *a*
*a*, *b*, *c* ()	12.047(3), 4.4525(11), 11.023(3)	12.056(3), 4.4519(11), 11.016(3)
*V* (^3^)	591.3(3)	591.2(3)
*Z*	4	4
Radiation type	Neutron, = 0.48-7.0	Neutron, = 0.48-7.0
(mm^1^)	0.00	0.00
Crystal size (mm)	4.00 4.00 4.00	4.00 4.00 4.00

Data collection		
Diffractometer	SXD	SXD
Absorption correction		
No. of measured, independent and observed [*I* > 2(*I*)] reflections	12888, 2773, 12888	7209, 1618, 7209

Refinement		
*R*[*F* ^2^ > 2(*F* ^2^)], *wR*(*F* ^2^), *S*	0.091, 0.259, 1.08	0.094, 0.266, 1.09
*R*_1_	0.0742	0.0713
*R*	0.0621	0.0585
No. of reflections	12888	7209
No. of parameters	104	104
No. of restraints	0	0
_max_, _min_ (e ^3^)	4.02, 5.60	2.09, 2.52
Extinction coefficient	0.048(3)	0.043(3)

For completeness, we report the Rietveld *R*-factors on *F* ^2^ (*R* and *wR*, where the weighting, *w*, is detailed in the CIFs) as well as the calculated *R*-factor based on *F* (*R*_1_), after the reflection set is merged for Fourier-map generation, and a measure of the signal to noise ratio in the data, *R* . Note that, whilst the values of *wR* are large and might be deemed unacceptable for an X-ray analysis, these are typical of single-crystal neutron refinements on *F* ^2^. Computer programs: *SXD*2001 (Gutmann, 2005 ▸), *SHELXS*2014 (Gruene *et al.*, 2014 ▸), *DIAMOND* (Putz Brandenburg, 2006 ▸), *publCIF* (Westrip, 2010 ▸).

**Table 2:** Comparison of covalent bond lengths (, uncorrected for thermal motion) obtained from DFT calculations and single-crystal neutron diffraction at 10 and 100K

	*Ab initio* (0K)	10K	100K
N1H1	1.0479	1.040(3)	1.040(6)
N1H2	1.0577	1.053(6)	1.053(8)
N1H3	1.0552	1.045(5)	1.048(8)
N2H4	1.0452	1.040(3)	1.037(6)
N2H5	1.0566	1.041(6)	1.043(8)
N2H6	1.0492	1.043(6)	1.039(8)
**Mean NH**	**1.051(5)**	**1.043(5)**	**1.042(5)**
O*w*1H7	0.9906	0.975(4)	0.972(6)
C1O1	1.2975	1.287(3)	1.286(4)
C1O2	1.2993	1.287(3)	1.282(5)
C1O3	1.3008	1.289(2)	1.288(4)
**Mean CO**	**1.299(2)**	**1.288(1)**	**1.285(3)**

**Table 3:** Comparison of bond angles () obtained from DFT calculations and single-crystal neutron diffraction at 10 and 100K

	*Ab initio* (0K)	10K	100K
H1N1H1^i^	108.14	108.0(5)	107.6(7)
H1N1H2	110.75	110.7(3)	111.0(4)
H1N1H3	108.85	109.2(3)	109.1(4)
H2N1H3	109.46	109.0(5)	108.9(7)
H4N2H4^i^	109.34	109.7(4)	109.4(7)
H4N2H5	108.23	107.9(3)	108.1(5)
H4N2H6	109.85	110.1(3)	110.1(5)
H5N2H6	111.28	111.0(5)	111.0(7)
**Mean HNH**	**109(1)**	**109(1)**	**109(1)**
H7O*w*1H7^ii^	106.39	106.2(6)	105.7(9)
O1C1O2	120.21	120.3(2)	120.2(3)
O1C1O3	120.41	120.2(3)	120.3(3)
O2C1O3	119.38	119.5(2)	119.6(3)
**Mean OCO**	**120.0(5)**	**120.0(4)**	**120.0(4)**

Symmetry codes: (i) 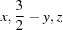; (ii) 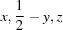.

**Table 4:** Hydrogen-bond lengths () and angles () determined by single-crystal neutron diffraction at 10 and 100K, and by DFT calculations

	*ab initio* (0K)			10K			100K		
	H*Y* ()	*X* *Y* ()	*X*H*Y* ()	H*Y* ()	*X* *Y* ()	*X*H*Y* ()	H*Y* ()	*X* *Y* ()	*X*H*Y* ()
N1H1O3	1.7640	2.8027	170.44	1.766(4)	2.797(2)	170.6(4)	1.764(6)	2.796(2)	170.7(6)
N1H2O1	1.7308	2.7819	171.89	1.721(6)	2.766(3)	171.1(5)	1.719(9)	2.765(4)	171.5(7)
N1H3O2	1.7436	2.7935	172.75	1.740(5)	2.778(2)	171.6(6)	1.739(8)	2.781(4)	171.8(8)
N2H4O1	1.8224	2.8604	171.58	1.808(3)	2.840(1)	171.7(4)	1.808(6)	2.838(2)	171.8(6)
N2H5O*w*1	1.7255	2.7745	171.32	1.807(7)	2.831(3)	167.0(5)	1.805(10)	2.830(5)	166.7(8)
N2H6O3	1.8130	2.8534	170.68	1.814(6)	2.847(3)	170.2(5)	1.816(8)	2.846(4)	170.4(8)
O*w*1H7O2	1.7937	2.7843	178.99	1.813(4)	2.788(2)	179.6(5)	1.816(6)	2.788(3)	179.8(7)

**Table 5:** Experimental and computational NH bond lengths () in ammonium ions in a variety of environments; crystallographic values are all sourced from single-crystal neutron diffraction studies Where there is more than one symmetry-inequivalent NH bond the mean bond length (and standard uncertainties) are reported.

Compound	Conditions	NH ()	Reference
NH_4_^+^	Gas phase	1.02873 0.00002	Crofton Oka (1987 ▸)
ND_4_^+^	Gas phase	1.0267 0.0005	Crofton Oka (1987 ▸)
NH_4_^+^	DFT (PW91)	1.055	Fortes *et al.* (2001 ▸)
NH_4_OH	DFT (PW91)	1.063 0.013	Fortes *et al.* (2001 ▸)
NH_4_CN	DFT (BLYP) I	1.034	Alavi *et al.* (2004 ▸)
NH_4_CN	DFT (BLYP) II	1.050	Alavi *et al.* (2004 ▸)
ND_4_(NH_3_)*_n_* clusters	(B3LYP/6-31+G*)	1.046 0.002	Wang *et al.* (2002 ▸)
ND_4_(NH_3_)*_n_* clusters	(MP2/6-31+G*)	1.043 0.003	Wang *et al.* (2002 ▸)
NH_4_Cl	295K	1.050 0.005	Kurki-Suonio *et al.* (1976 ▸)
NH_4_Br	296K	1.046 0.005	Seymour Pryor (1970 ▸)
NH_4_Br	409K	1.040 0.005	Seymour Pryor (1970 ▸)
NH_4_C_2_O_4_H_2_O	274K	1.029 0.005	Taylor Sabine (1972 ▸)
ND_4_C_2_O_4_D_2_O	274K	1.033 0.002	Taylor Sabine (1972 ▸)
(NH_4_)_2_C_4_H_4_O_6_	Room-temperature	1.04 0.03	Yadava Padmanabhan (1976 ▸)
(NH_4_)_2_SO_4_ (*Pnma*)	Room-temperature	1.069 0.018	Schlemper Hamilton (1966 ▸)
(NH_4_)_2_SO_4_ (*Pna*2_1_)	180K	1.050 0.016	Schlemper Hamilton (1966 ▸)
(NH_4_)_2_BeF_4_ (*Pnma*)	200K	0.989 0.014	Srivastava *et al.* (1999 ▸)
(NH_4_)_2_BeF_4_ (*Pna*2_1_)	163K	1.005 0.015	Srivastava *et al.* (1999 ▸)
(NH_4_)_2_BeF_4_ (*Pna*2_1_)	20K	1.018 0.015	Srivastava *et al.* (1999 ▸)
NH_4_ClO_4_	78K	0.995 0.010	Choi *et al.* (1974 ▸)
NH_4_ClO_4_	10K	1.037 0.014	Choi *et al.* (1974 ▸)
ND_4_NO_3_ (II)	355K	0.988 0.002	Lucas *et al.* (1979 ▸)
NH_4_NO_3_ (III)	298K	1.035 0.007	Choi Prask (1982 ▸)
NH_4_NO_3_ (IV)	298K	0.990 0.004	Choi *et al.* (1972 ▸)
ND_4_NO_3_ (IV)	298K	0.972 0.008	Lucas *et al.* (1979 ▸)
ND_4_NO_3_ (V)	233K	1.006 0.016	Ahtee *et al.* (1983 ▸)
NH_4_NH_3_CH_2_COOHSO_4_	Room-temperature	1.031 0.018	Vilminot *et al.* (1976 ▸)
(ND_4_)_2_Cu(SO_4_)_2_6D_2_O	15K	1.028 0.004	Simmons *et al.* (1993 ▸)
(ND_4_)_2_Cr(SO_4_)_2_6D_2_O	4.3K	1.028 0.002	Figgis *et al.* (1991 ▸)
(ND_4_)_2_Fe(SO_4_)_2_6D_2_O	4.3K	1.025 0.006	Figgis *et al.* (1989 ▸)
			
**(NH_4_)_2_CO_3_H_2_O**	**10K**	**1.043 0.005**	**This work**
	**100K**	**1.042 0.005**	
	**DFT (PBE)**	**1.051 0.005**	

**Table 6:** Properties of the electron density at the bond critical points in the ammonium ions as determined by DFT calculations Electron density, (*r*), is reported in e^3^, whereas the Laplacian, ^2^(*r*), and the eigenvalues of the Hessian matrix, _1_, _2_ and _3_, are given in e^5^.

	Fractional coordinates of BCP				Topology of electron density at BCP			
	*x*	*y*	*z*	(*r*)	^2^(*r*)	_1_	_2_	_3_
N1H1	0.1166	0.6071	0.0868	2.1581	47.083	29.697	28.538	11.152
N1H2	0.0344	0.7500	0.1360	2.0971	45.462	28.166	27.760	10.464
N1H3	0.1314	0.7500	0.1859	2.1145	46.979	28.915	28.472	10.407
N2H4	0.1299	0.8928	0.6928	2.1795	47.348	30.163	28.644	11.460
N2H5	0.0672	0.7500	0.6194	2.1038	48.144	29.216	28.682	9.754
N2H6	0.1748	0.7500	0.6052	2.1512	48.482	30.035	29.192	10.744
								
NH_4_F†		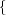	Expt.	1.975	13.0	22.3	21.6	31.0
			Calc.	1.865	11.2	21.1	20.2	30.3
NH_4_HF_2_ †		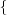	Expt.	2.230	35.0	33.8	31.3	30.1
			Calc.	2.185	28.3	29.2	28.9	29.7
NH_4_B_6_H_6_ ‡			Calc.	2.220	34.0	*29.6*	*29.3*	24.8
NH_4_C_2_HO_4_C_2_H_2_O_4_2H_2_O§			Expt.	2.207	37.0	30.1	29.3	22.4

† van Reeuwijk *et al.* (2000 ▸).

‡ Mebs *et al.* (2013 ▸) _1_ and _2_ estimated from the quoted bond ellipticity [ = _1_/ _2_) 1] and the Laplacian.

§ Stash *et al.* (2013 ▸).

**Table 7:** Properties of the electron density at the bond critical points in the hydrogen bonds as determined from the DFT calculations Electron density, (*r*), is reported in e^3^, whereas the Laplacian, ^2^(*r*), and the eigenvalues of the Hessian matrix, _1_, _2_ and _3_, are given in e^5^.

	Fractional coordinates of BCP			Topology of electron density at BCP				
	*x*	*y*	*z*	(*r*)	^2^(*r*)	_1_	_2_	_3_
H1O3	0.1429	0.4558	0.0492	0.2767	2.327	1.613	1.539	5.478
H2O1	0.0320	0.7500	0.1548	0.3124	2.543	1.874	1.825	6.242
H3O2	0.1615	0.7500	0.2539	0.2963	2.465	1.749	1.707	5.921
H4O1	0.1289	0.4531	0.7430	0.2460	2.124	1.362	1.317	4.803
H5O*w*1	0.0034	0.7500	0.5946	0.3158	2.572	1.933	1.854	6.360
H6O3	0.2333	0.7500	0.5602	0.2625	2.065	1.508	1.440	5.013
H7O2	0.1504	0.5358	0.3899	0.2561	2.079	1.464	1.414	4.957

**Table 8:** Energetic properties of the hydrogen bonds The local kinetic energy density, *G*(*r*), the local potential energy density, *V*(*r*), and the total energy density, *H*(*r*), at the bond critical point are all given in atomic units. Conversion factors used: 1a.u. of (*r*) = 6.7483e^3^; 1a.u. of ^2^(*r*) = 24.099e^5^; 1a.u. of energy density = 2625.4729kJmol^1^. The hydrogen bond energies, *E*_HB_, are in units of kJmol^1^.

	Derived hydrogen bond energy						
	*G*(*r*)	*V*(*r*)	*H*(*r*)	*E*_HB_ [equation (4)]	*E*_HB_ (corr)	*E*_HB_ [equation (5)]	*E*_HB_ mean
H1O3	0.03009	0.03604	0.00595	47.32	38.58	33.89	36.24
H2O1	0.03473	0.04307	0.00835	56.55	42.87	39.12	41.00
H3O2	0.03273	0.03989	0.00716	52.37	40.93	36.87	38.90
H4O1	0.02619	0.03035	0.00416	39.85	35.11	29.50	32.31
H5O*w*1	0.03524	0.04380	0.00856	57.50	43.32	39.69	41.50
H6O3	0.02711	0.03279	0.00568	43.04	36.59	30.53	33.56
H7O2	0.02669	0.03181	0.00512	41.75	36.00	30.06	33.03
**Mean**							**36.65**

**Table 9:** Assignment of observed Raman-active vibrational modes in ammonium carbonate monohydrate at 80K

Observed frequency (cm^1^)	Relative intensity (%)		Assignment	Observed frequency (cm^1^)	Relative intensity (%)		Assignment
175.2(4)	3.2			1935(1)	1.9	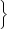	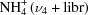
218.5(1)	42.9	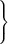	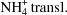	1990(2)	3.4		
234.1(1)	26.5			2037(4)	0.9		
258.6(1)	21.9			2185.9(6)	1.0	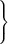	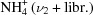
280.9(1)	16.6			2226.9(1)	5.9		
507(1)	2.0	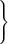	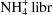	2263.1(2)	3.0		
528.0(4)	1.2			2296.5(5)	1.0		
573(2)	0.7			2683.0(3)	11.1	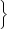	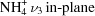
608(3)	0.4			2774.1(3)	7.5		
687.6(6)	3.0	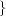	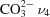	2839.8(3)	9.6		
709.2(6)	1.7			2889.5(2)	36.5		NH_4_^+^_1_
745.3(5)	3.7		H_2_O libr	2903(2)	15.6	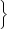	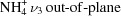
966(5)	1.4			2976(5)	12.4		
1056.8(7)	7.2		CO_3_^2^_1_(*B*_2*g*_)	3011(2)	16.0		
1074.66(4)	100.0		CO_3_^2^_1_(*A_g_*)	3066(3)	2.1		
1115(4)	1.3			3094(1)	10.4		
1384.8(2)	2.3		CO_3_^2^_3_(*A*_*g*_)	3134(2)	3.0		
1424.3(3)	2.7		CO_3_^2^_3_(*B*_2*g*_)	3224(1)	2.8		NH_4_^+^ (_2_ + _4_)?
1474.9(2)	3.5	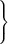	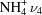	3296.88(4)	43.9		H_2_O _1_/_3_
1497.8(1)	5.0			3357(1)	4.4	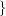	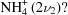
1517.5(4)	1.2			3494(1)	1.8		
1555.0(2)	2.9						
1696.4(5)	2.0	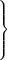	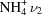				
1727.3(1)	8.1						
1745.2(2)	2.6						
1756.3(3)	2.9						
1769(1)	0.8						

**Table 10:** Unit-cell parameters of ammonium carbonate monohydrate as a function of temperature

	10K (neutron)	100K (neutron)	245K (X-ray)
*a* ()	12.047(3)	12.056(3)	12.160(7)
*b* ()	4.453(1)	4.452(1)	4.486(2)
*c* ()	11.023(3)	11.016(3)	11.011(7)
*V* (^3^)	591.3(3)	591.2(3)	600.7(6)

## Footnotes

1 Supporting information for this paper is available from the IUCr electronic archives (Reference: EB5035).
