# Supplementary material for: Structure, hydrogen bonding and thermal expansion of ammonium carbonate monohydrate
Source: Acta Crystallogr B Struct Sci Cryst Eng Mater. 2014 Dec 1;70(Pt 6):948–62. doi: 10.1107/S205252061402126X (PMC4468514; doi:10.1107/S205252061402126X)
Supplement: Supplementary file 6 [file b-70-00948-sup6.pdf]

SUPPLEMENTARY TABLES

Structure, hydrogen bonding, and thermal expansion of ammonium carbonate monohydrate

A. Dominic Fortes, Ian G. Wood, Dario Alfè, Eduardo R. Hernández, Matthias J. Gutmann, and Hazel A. Sparkes

*Corresponding author email: andrew.fortes@ucl.ac.uk

Supplementary Table S1

Experimentally-determined fractional atomic coordinates (*x*,*y*,*z*) and isotropic atomic displacement parameters (U_{iso}) at 10 K; values found by zero-pressure athermal DFT calculations are given in italics. Unit cell parameters at 10 K; *a* = 12.047(3) Å, *b* = 4.453(1) Å, *c* = 11.023(3) Å: unit cell parameters from the DFT calculations; *a* = 12.1408 Å, *b* = 4.4739 Å, *c* = 11.1552 Å.

Atom	*x*	*y*	*z*	Equiv U_{iso} (x10^3) $Å^2$
N1	0.0963(1)<br>*0.09915*	0.75	0.1220(1)<br>*0.12366*	5.1(2)
N2	0.1296(1)<br>*0.12601*	0.75	0.6413(1)<br>*0.65232*	5.0(2)
C1	0.3001(2)<br>*0.29712*	0.75	0.3813(1)<br>*0.38841*	2.4(2)
O1	0.3777(2)<br>*0.37775*	0.75	0.3011(2)<br>*0.31208*	4.4(3)
O2	0.1975(2)<br>*0.19580*	0.75	0.3485(2)<br>*0.35092*	4.6(3)
O3	0.3244(2)<br>*0.31684*	0.75	0.4952(2)<br>*0.50302*	4.1(3)
Ow1	0.0912(2)<br>*0.08630*	0.25	0.4468(2)<br>*0.43971*	6.5(3)
H1	0.1174(4)<br>*0.12233*	0.5610(8)<br>*0.56034*	0.0716(3)<br>*0.07465*	18.5(7)
H2	0.0110(5)<br>*0.01333*	0.75	0.1424(5)<br>*0.13996*	17.7(8)
H3	0.1414(6)<br>*0.14176*	0.75	0.2030(4)<br>*0.20611*	17.5(9)
H4	0.1331(4)<br>*0.13091*	0.5591(7)<br>*0.55941*	0.6954(3)<br>*0.70624*	18.1(6)
H5	0.0534(5)<br>*0.04854*	0.75	0.5965(5)<br>*0.60917*	18.0(8)
H6	0.1952(5)<br>*0.19061*	0.75	0.5796(5)<br>*0.58986*	17.8(8)
H7	0.1281(4)<br>*0.12600*	0.4250(7)<br>*0.42729*	0.4122(4)<br>*0.40867*	19.3(6)
